# Wernicke Encephalopathy Secondary to Hyperemesis Gravidarum in a 22-Year-Old Female Patient: A Case Report

**DOI:** 10.7759/cureus.45172

**Published:** 2023-09-13

**Authors:** Ryan Borgemenke, Samuel Borgemenke, Sharal Mall, Patrick Pagur

**Affiliations:** 1 Diagnostic Radiology, Kettering Health Network, Kettering, USA; 2 Diagnostic Radiology, Ohio University Heritage College of Osteopathic Medicine, Athens, USA

**Keywords:** acute metabolic encephalopathy, symmetric bilateral thalami hyperintensities, hyperemesis gravidarum, thiamine deficiency, wernicke encephalopathy

## Abstract

Wernicke encephalopathy is an acute, reversible neurological disorder, which, if untreated, can develop into Korsakoff syndrome. Commonly associated with alcohol use disorder, Wernicke encephalopathy is a metabolic disorder caused by a deficiency of thiamine, vitamin B1. This case report presents a clinical manifestation of Wernicke encephalopathy, in which a 22-year-old pregnant female with hyperemesis gravidarum and significant weight loss developed acute metabolic encephalopathy. Diagnostic imaging played an important role both in diagnosing the patient acutely in the hospital and during a follow-up visit one year after treatment.

## Introduction

Wernicke encephalopathy is an acute, reversible neurological disorder caused by a deficiency of thiamine, requiring urgent treatment to prevent severe neurological complications and death [1]. Thiamine, also known as vitamin B1, plays a vital role on a molecular level in the metabolism of carbohydrates and amino acids, which is why an absence of thiamine leads to metabolic abnormalities [2]. If left untreated, approximately 80% of Wernicke encephalopathy patients develop Korsakoff syndrome [3]. While Wernicke encephalopathy is commonly associated with alcohol use disorder, it is important to recognize that it can also occur in nonalcoholic patients, a fact often overlooked [1,4]. Strong indicators of nonalcoholic Wernicke-Korsakoff in adults are vomiting and significant weight loss [4], which can occur in hyperemesis gravidarum.

Hyperemesis gravidarum, characterized by severe nausea and vomiting during pregnancy, is a distressing complication affecting 1.5%-3.0% of pregnant women [1,5]. Hyperemesis gravidarum can persist throughout pregnancy, leading to malnutrition, dehydration, electrolyte imbalances, and unintentional weight loss [6]. Studies have shown that pregnant patients with Wernicke encephalopathy experience thiamine depletion between 10 and 15 weeks of gestation [7,8], vomit for a median duration of seven weeks, and lose an average of 12.1 kg [7]. Effective management of hyperemesis gravidarum requires a combination of medical interventions, lifestyle changes, dietary adjustments, supportive care, and patient education [6,7]. This case report presents an atypical clinical manifestation of Wernicke encephalopathy, with the aim of increasing awareness regarding potential complications associated with hyperemesis gravidarum. Furthermore, it seeks to promote patient care through early and accurate diagnosis.

## Case presentation

A 22-year-old pregnant female with a past medical history of asthma and polycystic ovarian syndrome presented to the emergency department with altered mental status, blurred vision, and dizziness. The patient was 13 weeks pregnant and G1P0. Of note, three days before coming to the emergency department, the patient was discharged after a five-day hospitalization for hyperemesis gravidarum. 

At that time, the patient had reported poor oral intake and 30 lbs weight loss in the first trimester of pregnancy due to excessive vomiting. The patient was found to have hypokalemia and transaminitis. Obstetrics and Gynecology was consulted. An acute hepatitis panel was ordered and was negative. A right upper quadrant ultrasound was performed and was unremarkable. The patient was treated with IV fluids, antiemetics, thiamine, and potassium supplementation. The Gastroenterology service was consulted and they felt that the transaminitis was secondary to vomiting. The patient was discharged with a peripherally inserted central catheter (PICC) line, ondansetron pump, and scheduled promethazine with a plan in addition to follow-up with Obstetrics and Gynecology.

When the patient presented to the emergency department just a few days after being hospitalized for hyperemesis gravidarum, the patient’s husband stated that the patient’s new symptoms began approximately 24 hours prior. The patient had no other relevant medical, surgical, or family history.

During the physical examination in the emergency department, the patient was disoriented and confused. The patient was alert and able to follow commands, but she was not interactive unless directly addressed and appeared to be in a “trance-like state,” staring at the ceiling. Mild scleral icterus was noted. A cardiovascular examination revealed a tachycardia with a regular rhythm. Lungs were clear to auscultation bilaterally, and the abdomen was soft, non-tender, and non-distended.

Initial lab work (Table 1), which included a complete blood count, showed mild anemia. White blood cell count was within normal limits. The basic metabolic panel showed hyponatremia. A liver profile panel showed transaminitis and hyperbilirubinemia, which was attributed to hyperemesis. Lactic acid and ammonia were also elevated. The serum tox screen was negative. Arterial blood gas (ABG) was within normal limits. Blood cultures were obtained and were negative.

**Table 1:** Initial blood work with tested value and reference range AST, aspartate transaminase; ALT, alanine transaminase.

	Tested Value	Reference Range
Hemoglobin	9.1 g/dL	12.1-15.8 g/dL
Sodium	133 mmol/L	134-144 mmol/L
AST	359 IU/L	0-40 IU/L
ALT	437 IU/L	0-32 IU/L
Alkaline Phosphatase	125 IU/L	44-121 IU/L
Direct Bilirubin	0.95 mg/dL	≤0.20 mg/dL
Total Bilirubin	2.1 mg/dL	0.3-1.0 mg/dL
Lactic Acid	3.5 mmol/L	0.5-2.0 mmol/L
Ammonia	58 umoL/L	6-47 umol/L

A head CT without contrast was obtained in the emergency department to exclude stroke. The head CT was unremarkable and there was no evidence of acute hemorrhage, infarct, or mass lesion. A right upper quadrant ultrasound was also unremarkable. The patient was treated with IV fluids, an ondansetron pump, and scheduled promethazine. The patient was subsequently admitted to the hospital for further evaluation and management. The patient’s tachycardia resolved. A repeat lactic acid value decreased back to within normal limits. The facility the patient was admitted to did not have inpatient Obstetrics and Gynecology available, so the patient was transferred to a hospital that did - the same facility as the last admission.

**Figure 1:**
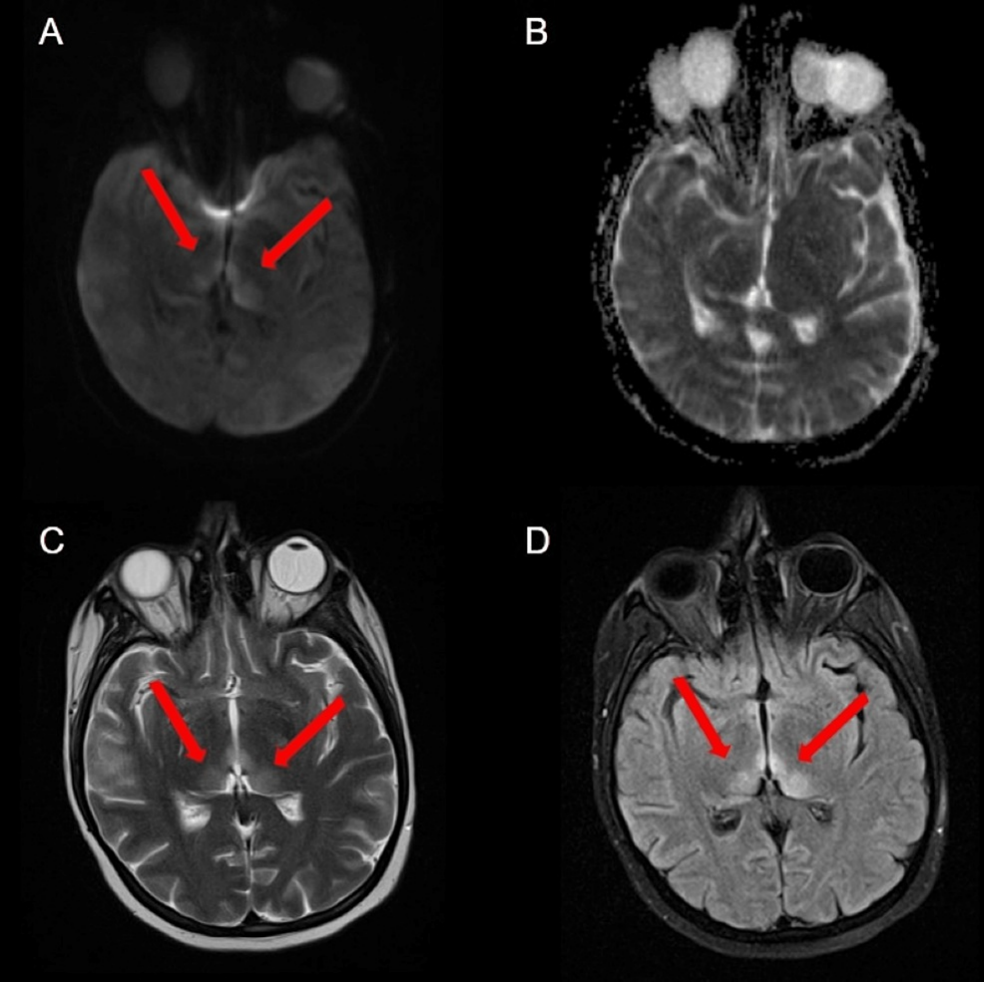
MRI brain without contrast MRI brain without contrast showing abnormal symmetric hyperintensities within the bilateral thalami (red arrows) displayed on axial DWI (A), axial T2 (C), and axial FLAIR (D). There is no increased signal in the bilateral thalami on ADC sequence (B), indicating diffusion restriction in the bilateral thalami. DWI, diffusion-weighted imaging; FLAIR, fluid-attenuated inversion recovery; ADC, apparent diffusion coefficient.

After the patient transfer, an MRI of the brain without contrast was obtained to further investigate the acute metabolic encephalopathy. The examination was slightly compromised due to motion artifact. The brain MRI demonstrated abnormal symmetric hyperintensities within the bilateral thalami (Figure 1A, 1C, 1D). This was felt to be secondary to Wernicke encephalopathy, in the setting of the patient’s known history, with variant Creutzfeldt-Jakob disease and infarcts of the bilateral thalami felt to be less likely. There was no evidence of intracranial or extracranial fluid or blood collection or evidence of acute large vessel territory stroke.

The Neurology team was consulted and their complete neurologic exam demonstrated disorientation, mildly dysarthric speech, ophthalmoplegia with poor extraocular movement, and no nystagmus. The remainder of the cranial nerves were intact. Rapidly alternating movements were slow and the patient had mild dysmetria bilaterally with the finger-to-nose test. Strength was 4+/5 in the bilateral upper extremities. Deep tendon reflexes were 2+/4 biceps, brachioradialis, patellar, and Achilles bilaterally. The gait exam was deferred, but nursing and occupational therapy notes endorsed overall delayed gross motor movements in the upper and lower extremities with increased time and attention for all tasks, including finger-to-nose assessment. The summation of the patient’s symptoms, image findings, and lab abnormalities led to the diagnosis of Wernicke encephalopathy. The patient was started on IV thiamine 500 mg three times daily. When other B vitamin levels were checked, the patient was found to have low folate (vitamin B9) 3.28 ng/mL (reference range 5.90-24.80 ng/mL) and a normal B12 level. The patient was continued on IV thiamine and the patient's mental status improved over the course of her admission. The patient’s scleral icterus resolved and her lab values returned to within normal limits. The Nutrition team was also consulted during the hospitalization. After five days, the patient was able to tolerate oral intake again, so she was transitioned to oral thiamine 100 mg daily. The patient was subsequently discharged home with a follow-up scheduled with her obstetrician and neurologist. 

**Figure 2:**
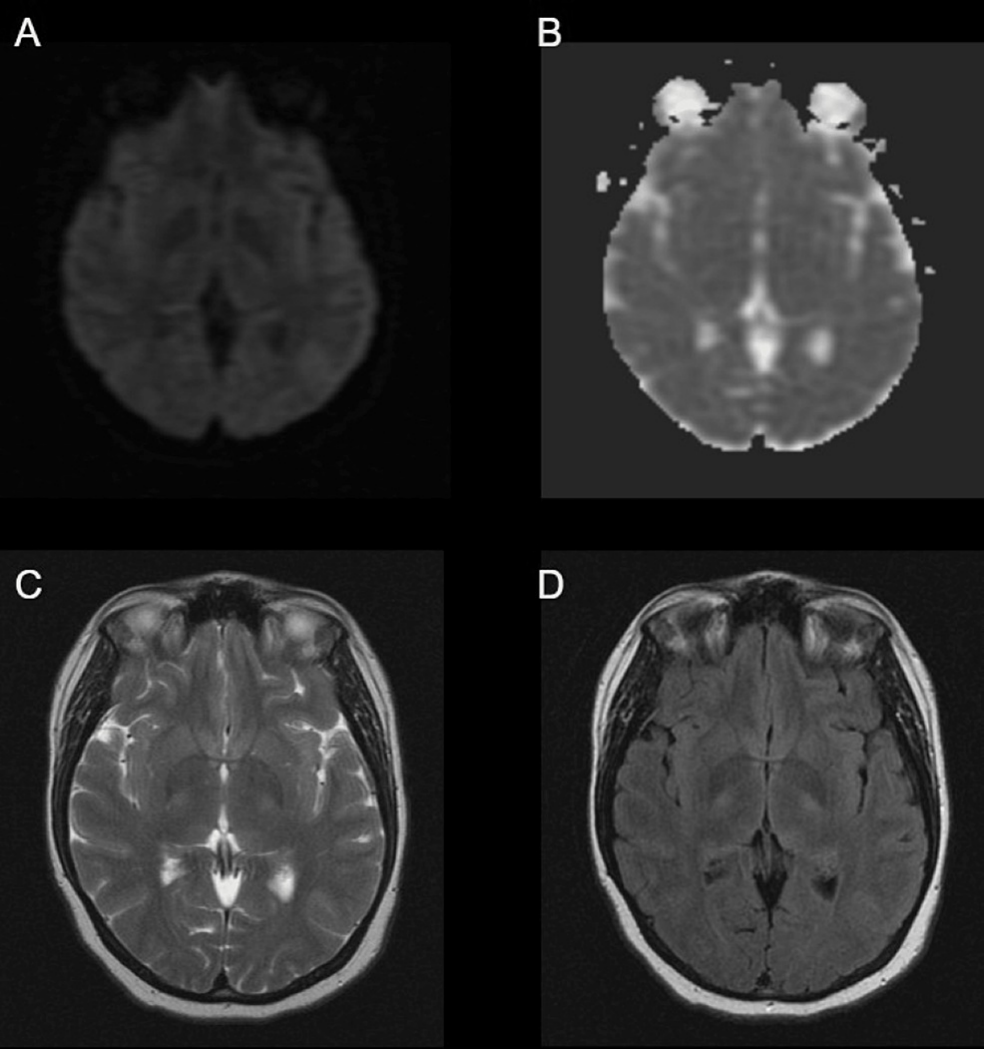
MRI brain without contrast one year after treatment MRI brain without contrast showing complete interval resolution of the abnormal hyperintensities within the bilateral thalami on axial DWI, T2, and FLAIR sequences (A, C, D). There was no abnormal signal on the axial ADC sequence (B). No evidence of diffusion restriction. DWI, diffusion-weighted imaging; FLAIR, fluid-attenuated inversion recovery; ADC, apparent diffusion coefficient.

Approximately one year after her hospital admission, the patient’s neurologist ordered a brain MRI. The patient’s brain MRI one year after treatment demonstrated complete interval resolution of the abnormal hyperintensities within the bilateral thalami (Figure 2A, 2C, 2D). There was no abnormal signal on the axial apparent diffusion coefficient (ADC) sequence (Figure 2B). No other abnormality was identified and there was no evidence of diffusion restriction.

## Discussion

This case report highlights a clinical presentation of Wernicke encephalopathy in a pregnant patient with hyperemesis gravidarum, emphasizing the importance of timely diagnosis and treatment to prevent further complications. The presented case emphasizes the complexity of diagnosing Wernicke encephalopathy, especially when the presentation differs from the classic triad of symptoms (confusion, ataxia, and nystagmus) [3,9]. Although they are often taught during medical school and tested on board examinations, the classic triad of symptoms is often not present [3,4]. The patient presented in this case did not have symptoms of nystagmus. This reinforces the need for a high index of suspicion and thorough clinical evaluation, even in the absence of the hallmark symptoms, particularly in patients with risk factors such as hyperemesis gravidarum and significant weight loss.

Hyperemesis gravidarum is a distressing complication of pregnancy characterized by severe nausea and vomiting. The presented case illustrates the severity of hyperemesis gravidarum, where the patient experienced poor oral intake and substantial weight loss, which led to significant malnutrition, dehydration, and electrolyte/vitamin abnormalities. After enough time, the patient had neurological manifestations of her vitamin B1 deficiency.

This case underscores the value of diagnostic imaging, particularly in cases of acute encephalopathy. The differential diagnosis for acute encephalopathy is quite expansive. One popular mnemonic for causes of acute encephalopathy is “AEIOU TIPS,” which stands for Alcohol, Epilepsy/Electrolytes/Encephalopathy, Insulin (hyperglycemia/hypoglycemia), Opiates/Oxygen, Uremia, Trauma/Temperature (hyperthermia/hypothermia), Infection, Poisons/Psychogenic, and Stroke/Seizures. While ruling out stroke (via negative head CT), infection, and toxins, the emergency department discovered clinical history and lab abnormalities which led to diagnoses involving malnutrition. The brain MRI was key because it revealed abnormal symmetric hyperintensities within the bilateral thalami on fluid-attenuated inversion recovery (FLAIR) sequence, specifically within the pulvinar thalamic nuclei. The so-called “hockey stick sign” can also be found in variant Creutzfeldt-Jakob disease and infarcts of the bilateral thalami. However, given the patient’s history and clinical presentation, the finding is highly suggestive of Wernicke encephalopathy.

Effective management of Wernicke encephalopathy entails timely and appropriate thiamine supplementation [10]. In this case, the patient received IV thiamine followed by oral supplementation, leading to a significant improvement in mental status and as well as documented radiographic improvement on brain MRI one year after treatment. The follow-up brain MRI showed complete interval resolution of abnormal bilateral thalamic hyperintensities. The favorable outcome in this case further supports the efficacy of rapid thiamine replacement therapy in reversing neurological deficits associated with thiamine deficiency, before permanent neurological damage and Korsakoff syndrome occur.

The presented case also highlights the need for a multidisciplinary approach to patient care. The diagnosis of Wernicke encephalopathy was achieved via interdepartmental collaboration of Emergency Medicine, Internal Medicine, Radiology, Obstetrics and Gynecology, Neurology, and Nutrition. Members of all teams ensured comprehensive management which was tailored to the patient's needs. The collaborative effort among healthcare providers contributed to the patient's successful recovery and underscored the importance of holistic patient-centered care.

## Conclusions

The presented case highlights the critical significance of timely recognition and intervention of Wernicke encephalopathy. The complexity of diagnosing Wernicke encephalopathy in the absence of the classic triad of symptoms underscores the need for a thorough clinical history and assessment as well as a high index of suspicion, particularly in patients with risk factors such as hyperemesis gravidarum. Diagnostic imaging was vital in achieving the diagnosis of Wernicke encephalopathy because the differential diagnosis of acute encephalopathy is broad. The successful outcome achieved in this case was also accomplished through multidisciplinary collaboration and prompt thiamine supplementation. This case report contributes to increasing awareness among healthcare providers about the potential risks and complications associated with thiamine deficiency during pregnancy, ultimately guiding clinical practice and enhancing patient care.
